# Supplementary material for: Choroidal and retinal thinning in chronic kidney disease independently associate with eGFR decline and are modifiable with treatment
Source: Nat Commun. 2023 Dec 5;14:7720. doi: 10.1038/s41467-023-43125-1 (PMC10697963; doi:10.1038/s41467-023-43125-1)
Supplement: Supplementary file 1 — Supplementary Information [file 41467_2023_43125_MOESM1_ESM.pdf]

Supplementary Material

Choroidal and retinal thinning in chronic kidney disease independently associate with eGFR decline and are modifiable with treatment

Tariq E Farrah[1,2]

Dan Pugh[1,2]

Fiona A Chapman[1,2]

Emily Godden[1,2]

Craig Balmforth[1]

Gabriel Oniscu[3,4]

David J Webb[1]

Baljean Dhillon[5,6]

Matthew A Bailey[1]

James W Dear[1]

Peter J Gallacher[1]

Neeraj Dhaun[1,2]

[1]University/BHF Centre for Cardiovascular Science, The Queen's Medical Research Institute, University of Edinburgh, Edinburgh, UK.

[2]Department of Renal Medicine, Royal Infirmary of Edinburgh, Edinburgh, UK

[3]Edinburgh Transplant Centre, Royal Infirmary of Edinburgh, Edinburgh, UK

[4]Transplant Division, Karolinska Institutet Stockholm, Sweden

[5]Centre for Clinical Brain Sciences, University of Edinburgh, Edinburgh, UK

[6]Princess Alexandra Eye Pavilion, Edinburgh, UK

Correspondence to: Dr Neeraj Dhaun *(Bean)*
University/BHF Centre for Cardiovascular Science
Queen's Medical Research Institute
47 Little France Crescent, Edinburgh.
E-mail: bean.dhaun@ed.ac.uk

Short title: OCT & the kidney

Contents

1 *Supplementary figure legends* ***Page 3***

2 *Supplementary tables* ***Page** 7*

Supplementary figures

Supplementary figure 1.

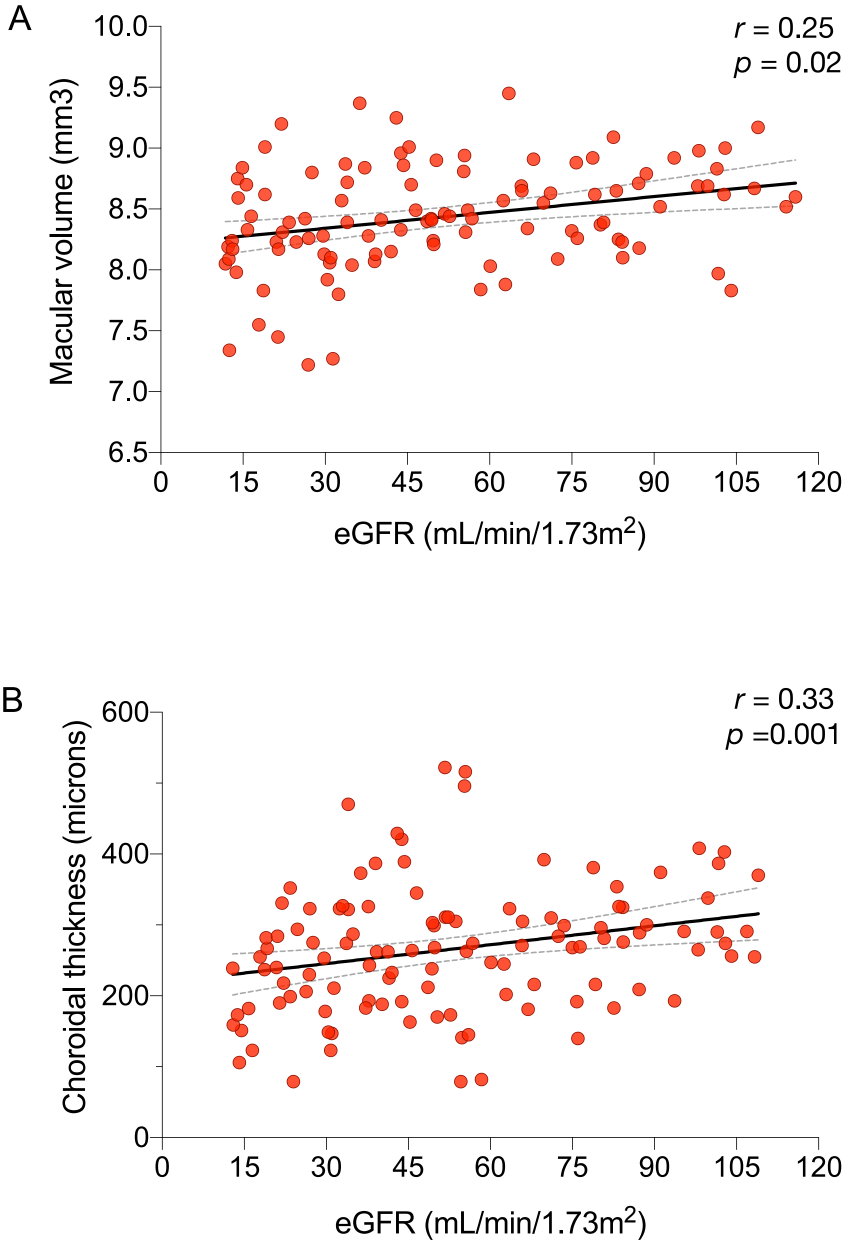

Supplementary figure 1. *OCT metrics and severity of kidney disease.* Scatter dot plots of estimated glomerular filtration rate (eGFR) and macular volume (**A**) and subfoveal choroidal thickness (**B**), n=112). Analysis by two-sided Pearson correlation coefficients.

Supplementary figure 2.

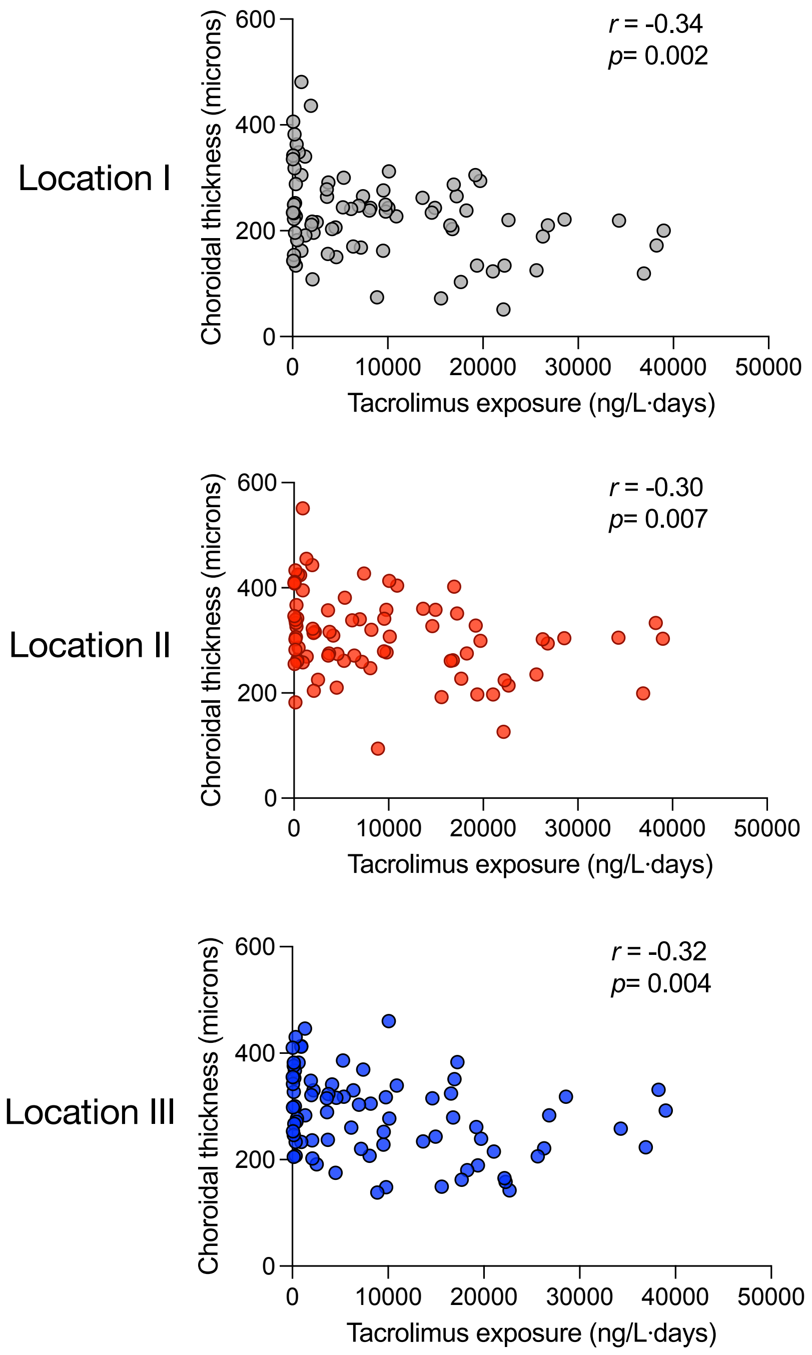

Supplementary figure 2. *Choroidal thickness and tacrolimus exposure.* Scatter dot plots of tacrolimus burden and choroidal thickness at location I (upper panel) and location II (middle panel) and location III (lower panel), n=91. Analysis by two-sided Spearman correlation coefficients.

Supplementary figure 3.

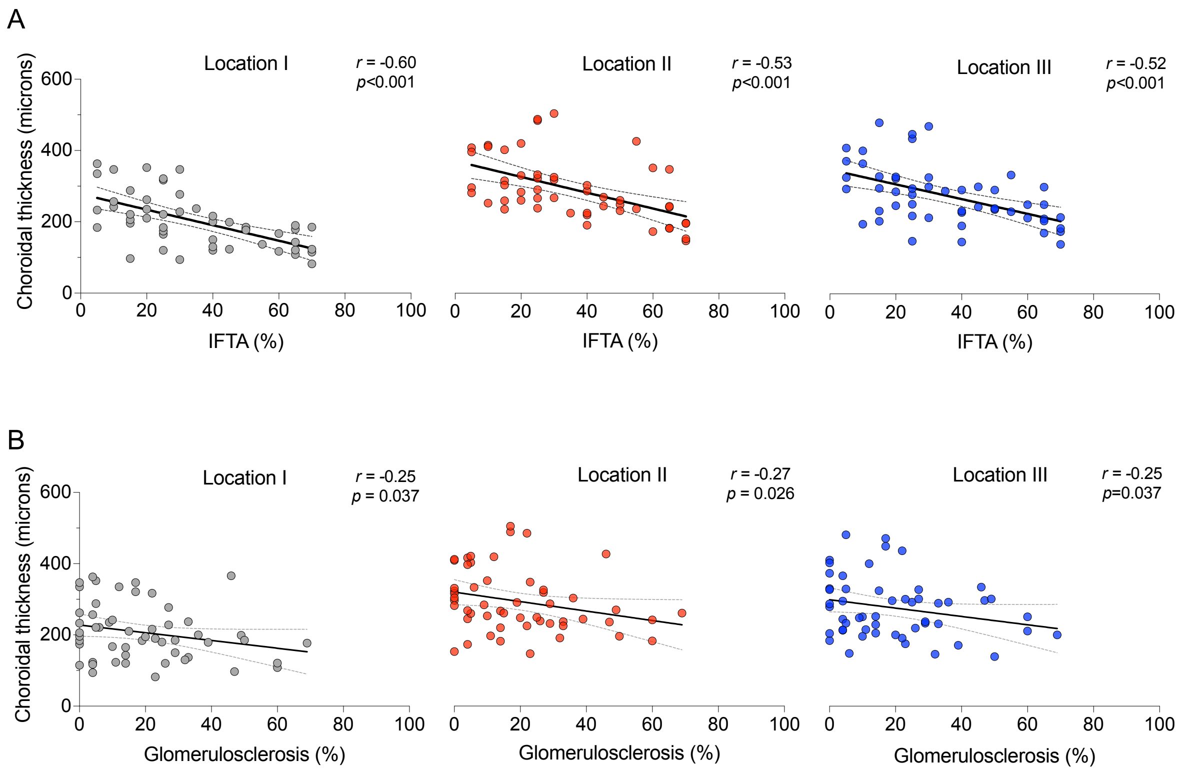

Supplementary figure 3. *Choroidal thickness and kidney histology.* Scatter dot plots of OCT-derived choroidal thickness at location I (left panels), location II (centre panels) and location III (right panels) in relation to extent of interstitial fibrosis and tubular atrophy (IFTA, **A**) and glomerulosclerosis (**B**) from paired native kidney biopsies, n=46. Analysis by two-sided Pearson correlation coefficients.

Supplementary figure 4.

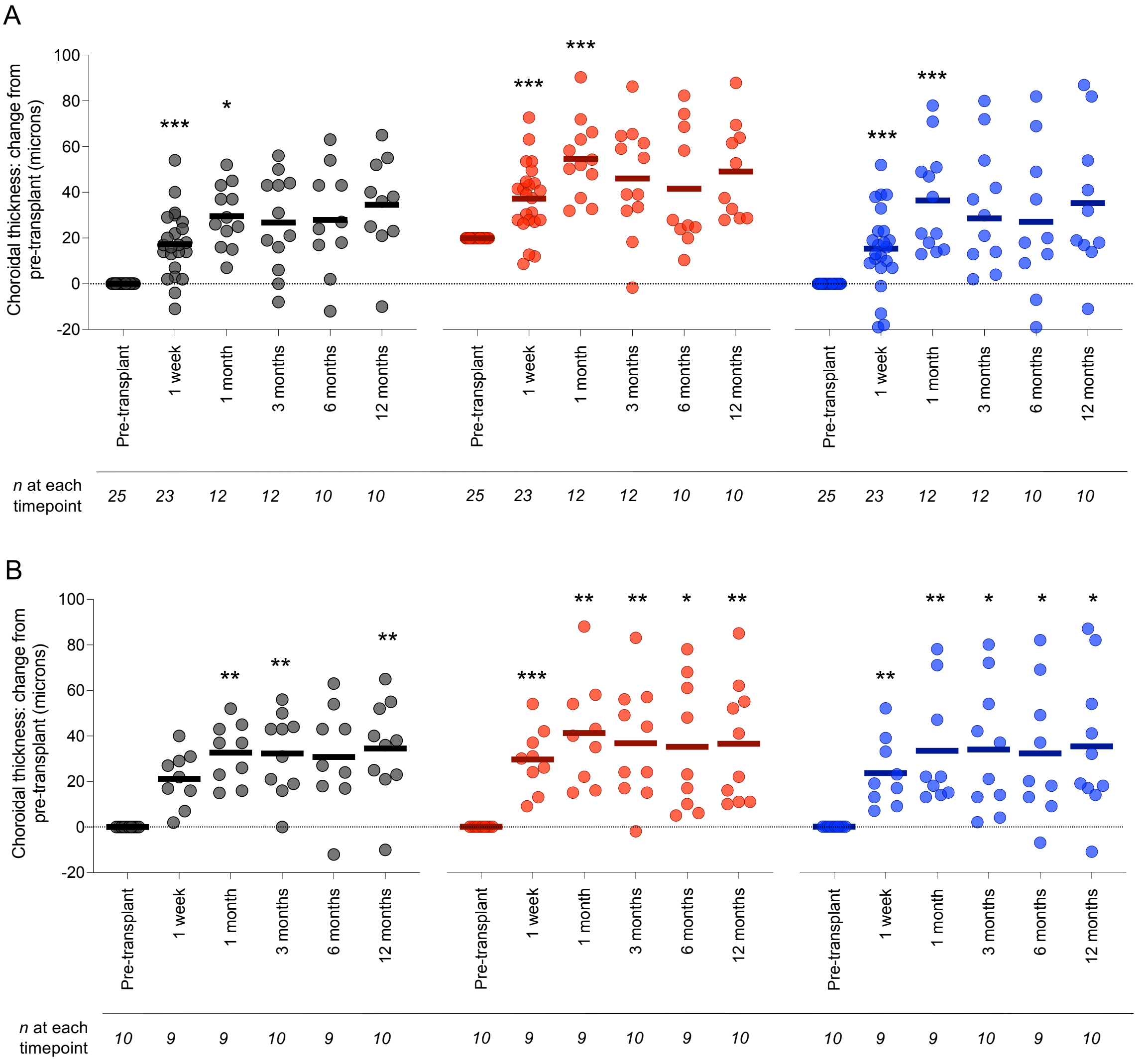

Supplementary figure 4. *Change in choroidal thickness following kidney transplantation.* Dot plot of change in pre-transplant choroidal thickness at 1 week, 1, 3, 6 and 12 months of participants with at least 1 follow up scan after living donor kidney transplantation (**A**) and restricted to participants with follow up scan at 12 months (**B**). Lines represent mean. Grey – choroidal thickness 2mm nasal to fovea; red – subfoveal choroidal thickness; blue – choroidal thickness 2mm temporal to fovea. **A.** Location I *vs.* pre transplant ***$p<0.001$ at 1 week, * $p=0.030$; Location II *vs.* pre transplant ***$p<0.001$ at 1 week and 1 month; Location III *vs.* pre transplant ***$p<0.001$ at 1 week, **$p=0.003$ at 1 month. **B.** Location I *vs.* pre transplant **$p=0.004$ at 1 month, **$p=0.001$ at 3 months, **$p=0.003$ at 12 months; Location II *vs.* pre

transplant ***$p<0.001$ at 1 week, **$p=0.002$ at 1 month, **$p=0.007$ at 3 months, *$p=0.013$ at 6 months, **$p=0.009$ at 12 months; Location III *vs.* pre transplant, **$p=0.003$ at 1 week, **$p=0.009$ at 1 month, *$p=0.019$ at 3 months, *$p=0.022$ at 6 months, ***$p<0.001$ at 1 week, *$p=0.030$ at 12 months. Two-sided analysis by mixed-effects model with Sidak correction for multiple comparisons.

Supplementary figure 5.

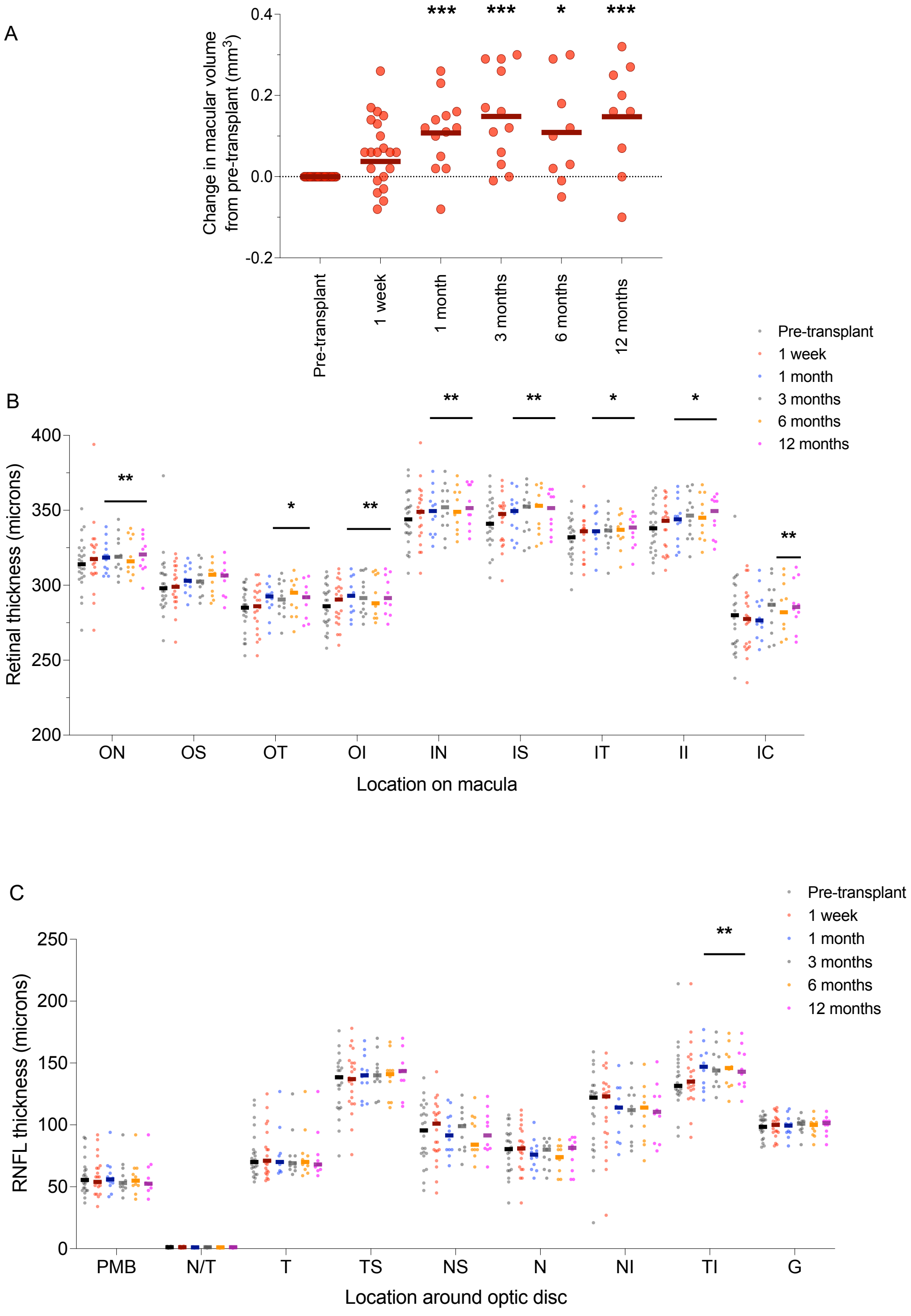

Supplementary figure 5. *Change in macular volume, retinal thickness and nerve fibre layer (RNFL) thickness following kidney transplantation.* Dot plot of change in pre-transplant macular volume (**A**), retinal thickness (**B**) and RNFL thickness (**C**) at 1 week, 1, 3, 6 and 12 months after living donor kidney transplantation. ON, outer nasal; OS, outer superior; OT, outer

temporal; OI, outer inferior; IN, inner nasal; IS, inner superior; IT, inner temporal; II, inner inferior; IC, inner circle. T, temporal; TS, temporal-superior; NS, nasal-superior; N, nasal; NI, nasal-inferior; TI, temporal-inferior; PMB, papillo-macular bundle; N/T, nasal-temporal ratio; G, average RNFL thickness. Lines represent mean. **A**. Macular volume *vs.* pre transplant ***$p<0.001$ at 1 month, 3 months. 12 months, *$p=0.016$ at 6 months. B. *$p<0.05$, **$p<0.01$, *** $p<0.001$ *vs.* pre-transplant. Analysis by two-sided mixed-effects model with Sidak correction for multiple comparisons.

Supplementary figure 6.

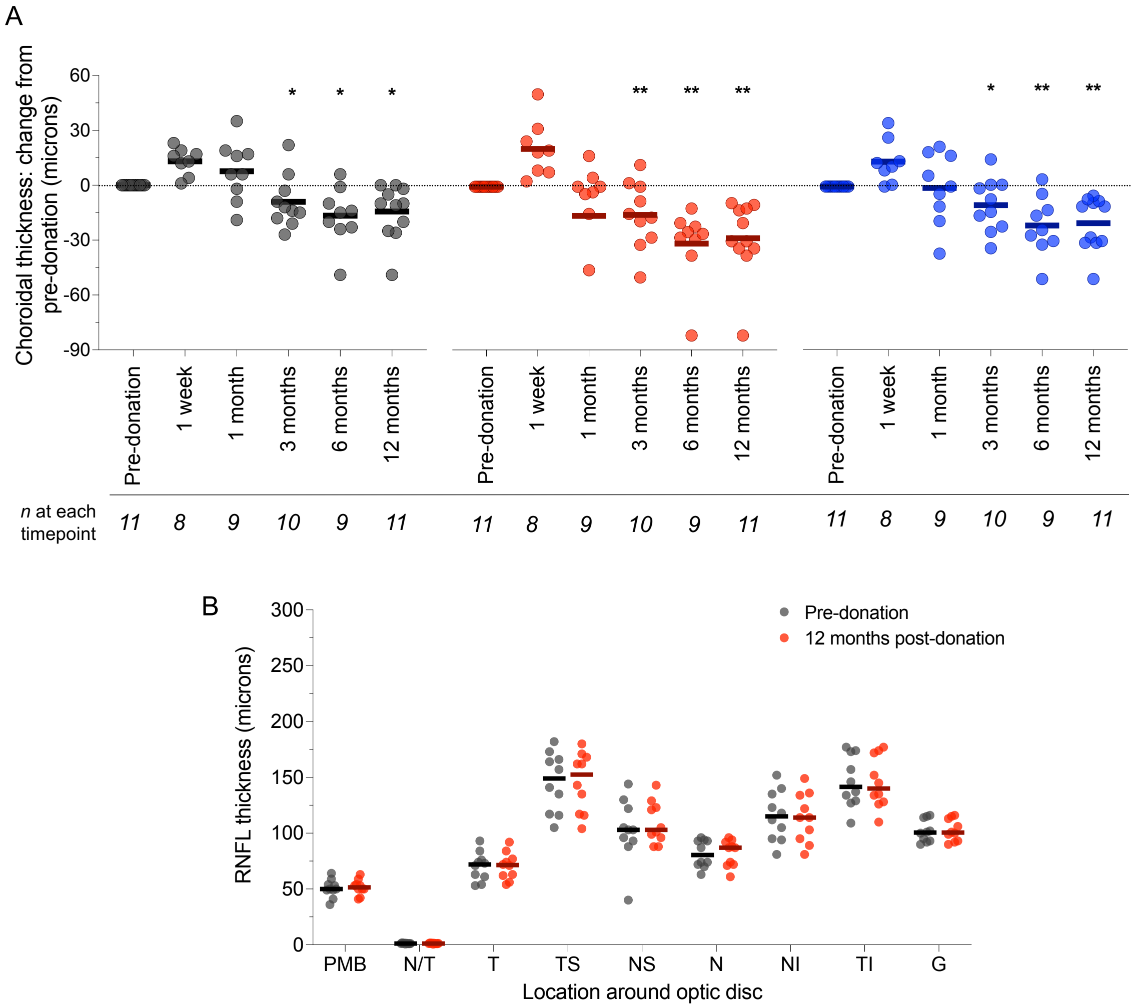

Supplementary figure 6. *Change in choroidal and nerve fibre layer thickness following donor nephrectomy.* Dot plot of change in pre-donation choroidal thickness at 1 week, 1, 3, 6 and 12 months after donor nephrectomy restricted to participants with a 12 month follow up scan (**A**) Grey – choroidal thickness 2mm nasal to fovea; red – subfoveal choroidal thickness; blue – choroidal thickness 2mm temporal to fovea. Dot plot of RNFL thickness at pre-donation and 12 months after nephrectomy (**B**). T, temporal; TS, temporal-superior; NS, nasal-superior; N, nasal; NI, nasal-inferior; TI, temporal-inferior; PMB, papillo-macular bundle; N/T, nasal-temporal ratio; G, average RNFL thickness. Lines represent mean. **A.** Location I *vs. pre-donation* **p*=0.041 at 3 months, **p*=0.012 at 6 months , **p*=0.040 at 12 months; Location II *vs. pre-donation* ***p*=0.010 at 3 months, ***p*=0.003 at 6 months, ***p*=0.004 at 12 months;

Location III *vs. pre-donation* *p=0.030 at 3 months, **p=0.005 at 6 months, **p=0.005 at 12 months. Two-sided analysis by mixed-effects model with Sidak correction for multiple comparisons.

Supplementary figure 7.

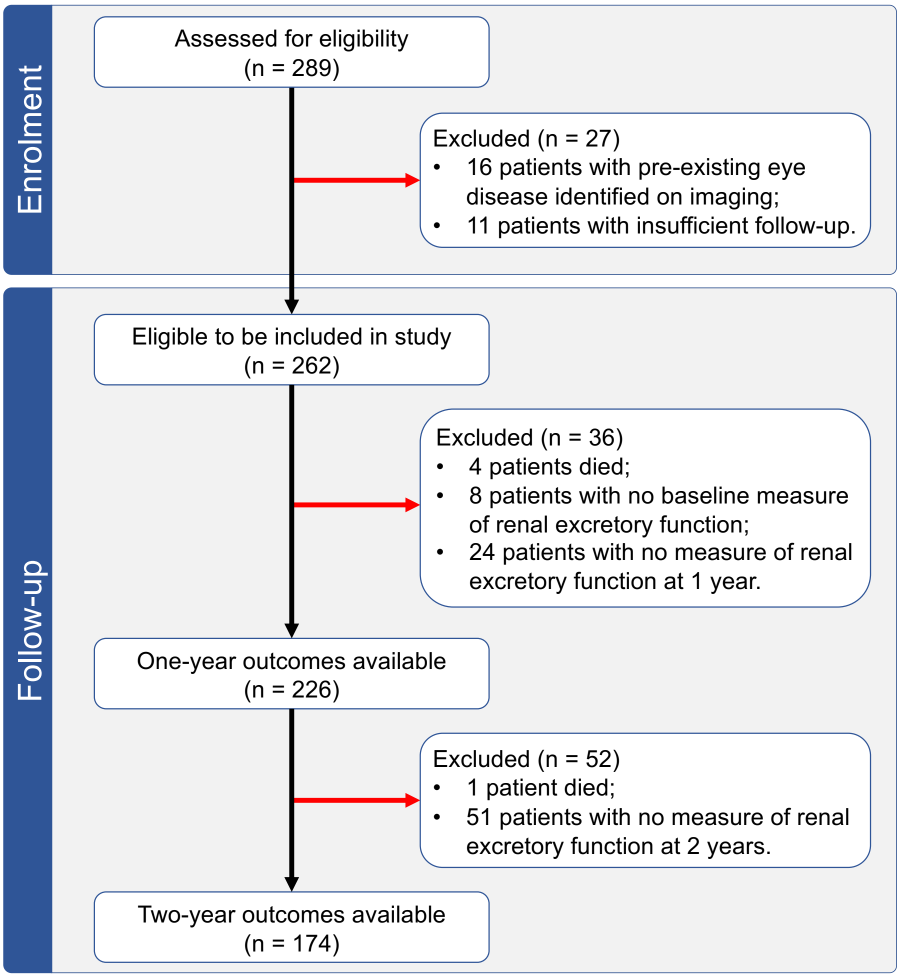

Supplementary figure 7. *STROBE diagram for analysis of OCT metrics and CKD progression*

Supplementary figure 8.

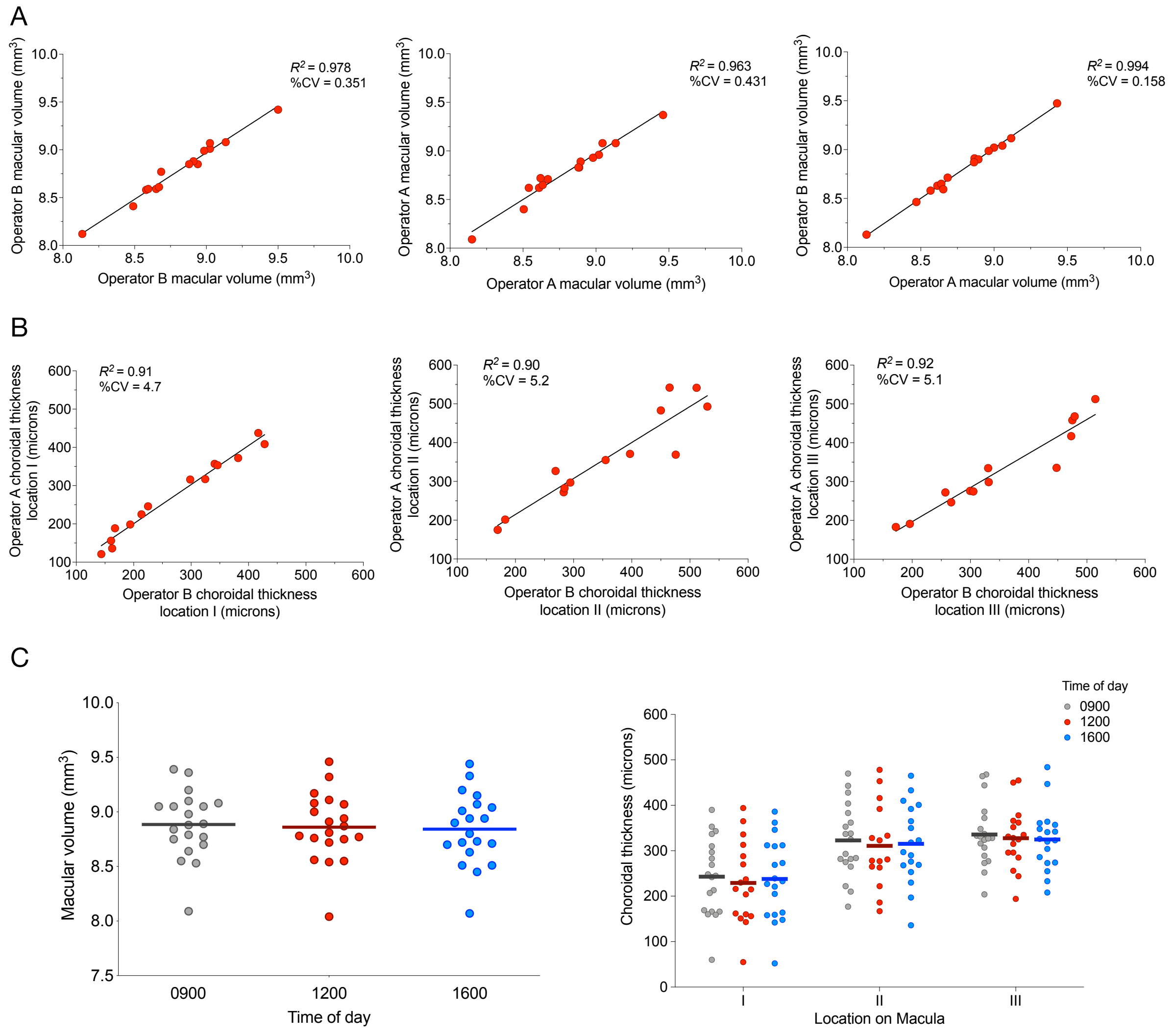

Supplementary figure 8. *Inter-, intra-operator and time of day variation* Scatter dot plots of intra-operator (left and centre panels) and inter-operator (right panel) variability for macular volume (**A**) in 15 healthy volunteers. Scatter dot plots of inter-operator variability for choroidal thickness (**B**) at location I (left panel), location II (centre panel) and location III (right panel) in 15 healthy volunteers. Scatter dot plots of macular volume (left panel) and choroidal thickness (right panel) from healthy volunteers who underwent OCT scanning at 0900, 1200 and 1600 (**C**). Line represents mean. CV – coefficient of variation, Analyses by linear regression, coefficient of variation and ANOVA.

Supplementary figure 9.

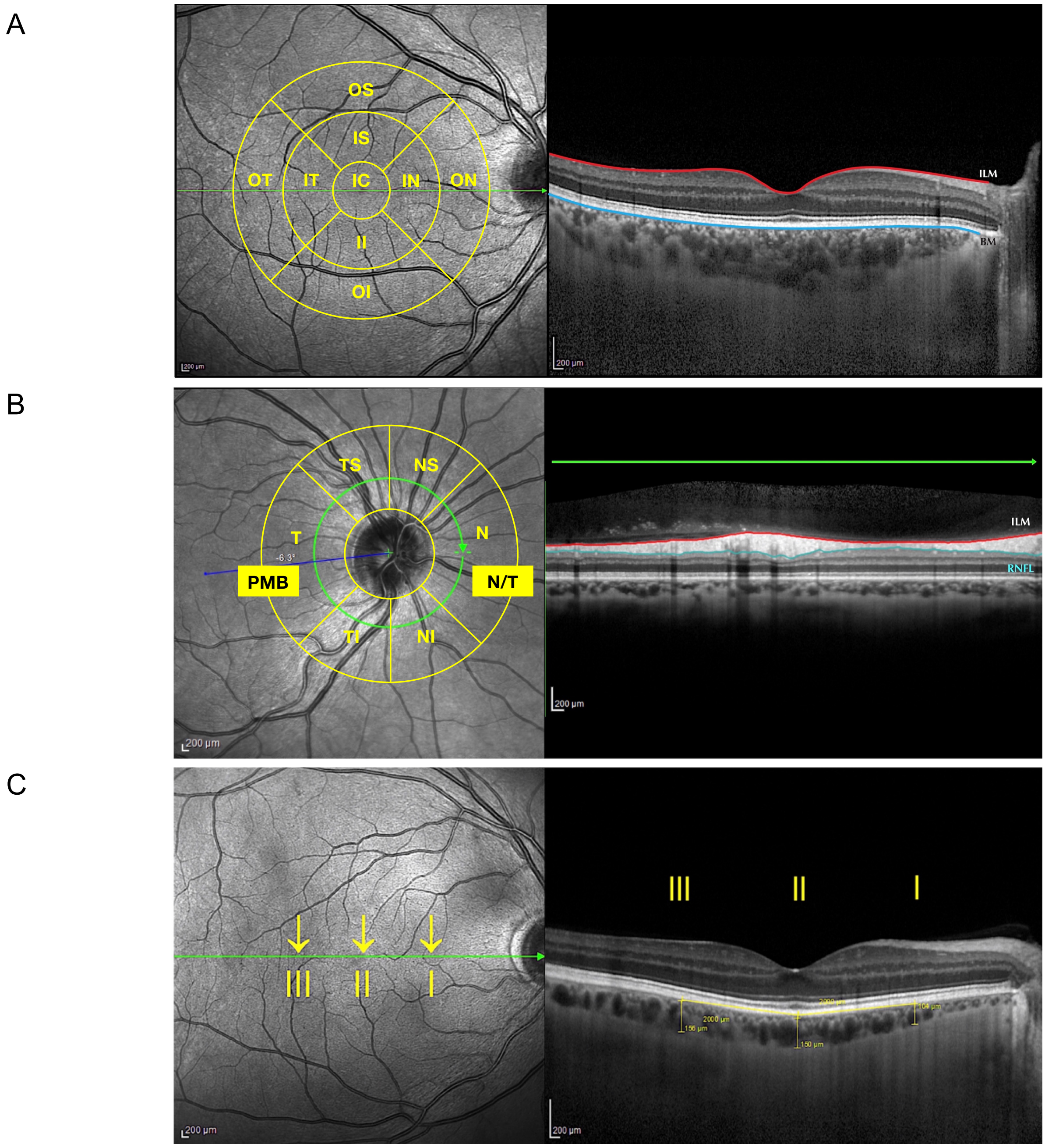

Supplementary figure 9. *OCT metrics.* Example images captured from the right eye by the Heidelberg SPECTRALIS® Spectral-Domain OCT (SD-OCT, Heidelberg Engineering, Heidelberg, Germany). **A.** *Left panel* shows a CLSO image centred over the right macula. Green line represents level and direction of cross section of corresponding OCT image. *Right panel* is a horizontal line OCT scan with the Early Treatment of Diabetic Retinopathy study map overlay. This divides the macula into 9 subfields. A circular grid is centred over the fovea and

consists of 3 concentric rings of diameters 1, 3, and 6mm, respectively. The inner and outer rings are further divided into quadrants: temporal, nasal, superior, and inferior. Retinal thickness is defined as the area bounded by internal limiting membrane (ILM, red) and Bruch's membrane (BM, blue). ON, outer nasal; OS, outer superior; OT, outer temporal; OI, outer inferior; IN, inner nasal; IS, inner superior; IT, inner temporal; II, inner inferior; IC, inner circle. **B.** *Left panel* is a CLSO image centred over the optic nerve head with line of cross-section (green) circled around the peri-papillary region. *Right panel* is an OCT image demonstrating retinal thickness from the circular cross-section around the optic nerve head in the left image. The green line running from left to right corresponds to the direction of cross-section of the green circle in left panel. Retinal nerve fibre layer (RNFL) thickness as defined by the red and cyan lines which is measured for 7 regions: T, temporal;TS, temporal-superior; NS, nasal-superior; N, nasal; NI, nasal-inferior; TI, temporal-inferior. PMB, papillo-macular bundle. Using these values, two additional measures are calculated: N/T, nasal-temporal ratio; G, average RNFL thickness. **C**. CSLO (*left panel*) and horizontal line OCT scan with Enhanced Depth Imaging (EDI, *right panel*). Choroidal thickness was defined as the distance between the outer hyper-reflective line of the retinal pigment epithelium (RPE) (RPE/basement membrane complex) to the choroidal-scleral junction and was measured at 3 locations: I = 2 mm nasal to the fovea, II = subfoveal, III = 2 mm temporal to the fovea as shown. The corresponding locations on the macula are indicated by yellow arrows.

Supplementary tables

Supplementary table 1. Linear regression model for predictors of macular volume in CKD patients, n=112. Table shows β coefficients expressed as change in macular volume atrophy per unit **increase** in the associated independent variable. *p* values <0.05 are in bold.

Variable	β (95% CI)	p
Age (per year)	-0.0015 (-0.0073 to 0.0064)	0.593
eGFR (per mL/min/1.73m$^{2)}$	0.0032 (0.0001 to 0.0063)	**0.042**

Model adjusted R^2 =0.5, F(2,109)=3.021, p=0.054
eGFR: estimated glomerular filtration rate

Supplementary table 2. *Linear regression model for predictors of subfoveal (location II) choroidal thickness in CKD patients, n=112. Table shows β coefficients expressed as change in choroidal thickness per unit* ***increase*** *in the associated independent variable.*

Variable	β (95% CI)	p
Age (per year)	-0.89 (-2.03 to 0.24)	0.120
eGFR (per mL/min/1.73m$^{2)}$	0.69 (0.10 to 1.29)	**0.021**

Model adjusted R^2 =0.10, F(2,110)=6.341, p=0.003
eGFR: estimated glomerular filtration rate.

Supplementary table 3. *Renal biopsy diagnoses*

Clinico-pathologic diagnosis	n (%)
ANCA-associated vasculitis	38 (76)
IgA nephropathy	5 (10)
Tubulointerstitial nephritis	3 (6)
Hypertensive nephrosclerosis	2 (4)
Focal segmental glomerulosclerosis	1 (2)
Lupus nephritis	1 (2)

ANCA: anti-neutrophil cytoplasm antibody, IgA: immunoglobulin A.

Supplementary table 4. Standard multiple linear regression models examining variables associated with interstitial fibrosis/tubular atrophy from kidney biopsy specimens from CKD patients (n=50). Tables show β coefficients expressed as change in interstitial fibrosis/tubular atrophy per unit **increase** in the associated independent variable, with associations of choroidal thickness at each location on the retina assessed separately. p values <0.05 are in bold.

Variable	β (95% CI)	p
Age (per year increase)	0.28 (-0.11 to 0.67)	0.150
eGFR (per ml/min/1.73m^2 increase)	-0.18 (-0.38 to 0.01)	0.063
Choroidal thickness at location I (per μm increase)	-0.11 (-0.20 to 0.03)	**0.009**
Glomerulosclerosis (per % increase)	0.28 (-0.02 to 0.58)	0.068
Mean arterial pressure (per 1 mmHg increase)	-0.11 (-0.62 to 0.41)	0.680
Pre-existing hypertension (presence)	0.34 (-10.78 to 11.35)	0.950

Model adjusted R^2=0.45, F(44,6)=5.49.1, p<0.001
eGFR: estimated glomerular filtration rate.

Variable	β (95% CI)	p
Age (per year increase)	0.29 (-0.12 to 0.71)	0.160
eGFR (per ml/min/1.73m^2 increase)	-0.17 (-0.38 to 0.03)	0.090
Choroidal thickness at location II (per μm increase)	-0.08 (-0.15 to 0.01)	**0.035**
Glomerulosclerosis (per % increase)	0.33 (0.02 to 0.62)	0.034
Mean arterial pressure (per 1 mmHg increase)	-0.07 (-0.62 to 0.49)	0.810
Pre-existing hypertension (presence)	0.12 (-11.16 to 11.41)	0.980

Model adjusted R^2=0.40, F(44,6)=5.49.1, p=0.001
eGFR: estimated glomerular filtration rate.

Variable	β (95% CI)	p
Age (per year increase)	0.27 (-0.15 to 0.69)	0.190
eGFR (per ml/min/1.73m^2 increase)	-0.15 (-0.34 to 0.05)	0.130
Choroidal thickness at location III (per μm increase)	-0.08 (-0.16 to 0.01)	**0.029**
Glomerulosclerosis (per % increase)	0.34 (-0.05 to 0.64)	0.023
Mean arterial pressure (per 1 mmHg increase)	-0.12 (-0.65 to 0.42)	0.660
Pre-existing hypertension (presence)	0.70 (-10.40 to 11.80)	0.902

Model adjusted R^2=0.40, F(44,6)=5.49.1, p=0.001
eGFR: estimated glomerular filtration rate.

Supplementary table 5. *Baseline characteristics of transplant recipients and kidney donors in study 3*

		Transplant recipients	Kidney donors
**Number of participants (n=47)**		25	22
**Age, years**		45 ± 14	50 ± 11
**Male, n (%)**		16 (62)	8 (36)
**Smoking status, n (%)**			
	Never	21 (81)	21 (95)
	Current	0 (0)	0 (0)
	Ex-smoker	5 (19)	1 (5)
**Primary renal diagnosis**			
	IgA nephropathy	9 (35)	
	Polycystic kidney disease	6 (23)	
	SLE	2 (8)	
	FSGS	2 (8)	
	Nephrolithiasis	1 (4)	
	Hypertension	1 (4)	
	Amyloid	1 (4)	
	Reflux nephropathy	1 (4)	
	HUS	1 (4)	
	Unknown	2 (8)	
**Clinical**			
BMI, kg/m^2		26.6 ± 4.2	26.8 ± 3.2
BP, mmHg			
	Systolic	135 ± 19	135 ± 15
	Diastolic	84 ± 9	86 ± 18
	MAP	87 ± 37	102 ± 16
**Laboratory**			
Creatinine, μmol/L		661 ± 201*	69 ± 8
eGFR, ml/min/1.73m^2		8 ± 3*	96 ± 9
GFR stage, ml/min/1.73m^2 n (%)			
	≥90	0	17 (77)
	60-89	0	5 (23)
	30-59	0	0
	15-29	0	0
	<15	26 (100)	0
Haemoglobin, g/L		114 ± 16	138 ± 9
hsCRP, mg/L		2 ± 3	1 ± 2
uPCR, mg/mmol		380 ± 393	3 ± 5
**Medications, n (%)**			
	Aspirin	0	0 (0)
	α-blocker	2 (8)	1 (5)
	ACE inhibitor	7 (27)	1 (5)
	Angiotensin receptor blocker	1 (4)	0 (0)
	β-blocker	4 (15)	0 (0)
	Calcium channel blocker	9 (35)	0 (0)
	Diuretic	2 (8)	0 (0)
	Statin	9 (35)	1 (5)

ACE: angiotensin converting enzyme, BMI: body mass index, BP: blood pressure, eGFR: estimated glomerular filtration rate, FSGS: focal and segmental glomerulosclerosis, HUS: haemolytic uraemic syndrome, hsCRP: high sensitivity C-reactive protein, MAP: mean arterial pressure, uPCR: urine protein:creatinine ratio, SLE: systemic lupus erythematosus. *Parameters influenced by dialysis.

Supplementary table 6. *Baseline characteristics for study 4 cohort*

**Number of participants**	**262**
**Age, years (SD)**	57 (14)
**Female, n (%)**	108 (41)
**Baseline BP, mmHg (SD)***	
Systolic	136 (19)
Diastolic	78 (12)
**Baseline serum creatinine, µmol/L (IQR)***	141 (103 – 227)
**Baseline eGFR, ml/min/1.73m^2 (IQR)***	42 (25 – 63)
**CKD stage, n (%)**	
1	14 (5)
2	57 (22)
3	106 (40)
4	52 (20)
5	25 (10)
**Baseline haemoglobin, g/L***	125 (19)
**Baseline uPCR, mg/mmol (IQR)***	51 (17 – 193)

Values are n (%), mean (standard deviation [SD]) or median (interquartile range [IQR]). Abbreviations: BP – blood pressure; eGFR – estimated glomerular filtration rate; uPCR – urinary protein:creatinine ratio. *Baseline BP data were available for 88% (231/262) of patients; baseline renal function data were available for 97% (254/262) of patients; baseline haemoglobin data were available for 77% (201/262) of patients; and baseline uPCR data were available for 88% (231/262) of patients.

Supplementary table 7. Summary of univariable logistic regression models evaluating relationship between each chorioretinal metric (total macular volume and choroidal thickness [locations one, two and three]) and the primary outcomes of a decline in eGFR of ≥10 at one year and ≥20% at two years. Two sided analyses. Abbreviations: CI – confidence interval; df – degrees of freedom; eGFR – estimated glomerular filtration rate; OR – odds ratio. *p* values <0.05 are in bold.

**Decline in eGFR of ≥10% at one year**							
	**Standard error**	**z-value**	**OR**	**Lower 95% CI**	**Upper 95% CI**	**p**	**Residual deviance**
**Total macular volume (per 1 mm^3 decrease)**	0.35	-2.57	2.48	1.26	5.08	**0.013**	298 on 220 df
**Choroid (location I) (per 10 µm decrease)**	0.002	-1.01	1.02	0.98	1.05	0.319	306 on 221 df
**Choroid (location II) (per 10 µm decrease)**	0.002	-1.76	1.03	1.00	1.06	0.081	304 on 221 df
**Choroid (location III) (per 10 µm decrease)**	0.002	-2.38	1.04	1.01	1.08	**0.024**	301 on 221 df
**Decline in eGFR of ≥20% at two years**							
	**Standard error**	**z-value**	**OR**	**Lower 95% CI**	**Upper 95% CI**	**p**	**Residual deviance**
**Total macular volume (per 1 mm^3 decrease)**	0.45	-2.91	3.75	1.59	9.54	**0.004**	211 on 170 df
**Choroid (location I) (per 10 µm decrease)**	0.002	-1.04	1.02	0.98	1.06	0.304	221 on 169 df
**Choroid (location II) (per 10 µm decrease)**	0.002	-1.29	1.02	0.99	1.06	0.209	220 on 169
**Choroid (location III) (per 10 µm decrease)**	0.002	-2.56	1.06	1.01	1.10	**0.012**	215 on 169

Supplementary table 8. Comparison of multivariable logistic regression models evaluating the relationship between total macular volume and the odds of an eGFR decline of ≥10% at one year. The table illustrates the effect of sequentially adding covariates to each logistic regression model. Based on its AIC and the likelihood ratio test statistic obtained when it was compared individually to the other five models, the model selected for the final analysis was Model 6. Please note: the odds ratios and 95% confidence intervals depicted for total macular volume refer to the effect of a 1 mm^3 *increase* in total macular volume, not a 1 mm^3 *decrease* – as is depicted elsewhere in the manuscript. Two sided analyses. Abbreviations: AIC – Akaike Information Criterion; BP – blood pressure; CI – confidence interval; eGFR – estimated glomerular filtration rate; PCR – protein:creatinine ratio. *p* values <0.05 are in bold.

	**Model 1**				**Model 2**				**Model 3**				**Model 4**				**Model 5**				**Model 6**			
Predictors	*Odds Ratios*	*std. Error*	*CI*	*p*	*Odds Ratios*	*std. Error*	*CI*	*p*	*Odds Ratios*	*std. Error*	*CI*	*p*	*Odds Ratios*	*std. Error*	*CI*	*p*	*Odds Ratios*	*std. Error*	*CI*	*p*	*Odds Ratios*	*std. Error*	*CI*	*p*
Total macular volume (per 1mm3 increase)	0.40	0.14	0.20 – 0.80	**0.010**	0.33	0.13	0.15 – 0.68	**0.003**	0.33	0.13	0.15 – 0.68	**0.003**	0.40	0.16	0.18 – 0.84	**0.019**	0.39	0.16	0.17 – 0.85	**0.020**	0.41	0.18	0.18 – 0.93	**0.038**
Age (per 1 year increase)					0.98	0.01	0.96 – 1.00	0.060	0.98	0.01	0.96 – 1.00	0.060	0.97	0.01	0.95 – 1.00	**0.019**	0.98	0.01	0.95 – 1.00	**0.035**	0.98	0.01	0.96 – 1.00	0.089
Female sex									0.95	0.27	0.55 – 1.65	0.865	0.95	0.28	0.54 – 1.69	0.873	1.09	0.33	0.61 – 1.97	0.766	0.95	0.30	0.51 – 1.77	0.869
Baseline eGFR (per 1ml/min increase)													0.98	0.01	0.96 – 0.99	**<0.001**	0.98	0.01	0.96 – 0.99	**<0.001**	0.98	0.01	0.96 – 0.99	**<0.001**
Baseline systolic BP (per 1mmHg increase)																	0.99	0.01	0.98 – 1.01	0.345	0.99	0.01	0.97 – 1.00	0.126
Baseline urinary PCR (per 1mg/mmol increase)																					1.00	0.00	1.00 – 1.01	**0.001**
AIC	302.220				300.609				302.580				288.803				277.188				254.002			
log-Likelihood	-149.110				-147.305				-147.290				-139.401				-132.594				-120.001			

Supplementary table 9. Comparison of multivariable logistic regression models evaluating the relationship between total macular volume and the odds of an eGFR decline of ≥20% at two years. The table illustrates the effect of sequentially adding covariates to each logistic regression model. Based on its AIC and the likelihood ratio test statistic obtained when it was compared individually to the other five models, the model selected for the final analysis was Model 6. Please note: the odds ratios and 95% confidence intervals depicted for total macular volume refer to the effect of a 1 mm^3 *increase* in total macular volume, not a 1 mm^3 *decrease* – as is depicted elsewhere in the manuscript. Two sided analyses. Abbreviations: AIC – Akaike Information Criterion; BP – blood pressure; CI – confidence interval; eGFR – estimated glomerular filtration rate; PCR – protein:creatinine ratio.

	**Model 1**				**Model 2**				**Model 3**				**Model 4**				**Model 5**				**Model 6**			
Predictors	*Odds Ratios*	*std. Error*	*CI*	*p*	*Odds Ratios*	*std. Error*	*CI*	*p*	*Odds Ratios*	*std. Error*	*CI*	*p*	*Odds Ratios*	*std. Error*	*CI*	*p*	*Odds Ratios*	*std. Error*	*CI*	*p*	*Odds Ratios*	*std. Error*	*CI*	*p*
Total macular volume (per 1mm3 increase)	0.27	0.12	0.10 – 0.63	**0.004**	0.26	0.12	0.10 – 0.63	**0.004**	0.26	0.12	0.10 – 0.63	**0.004**	0.27	0.13	0.10 – 0.67	**0.006**	0.31	0.15	0.12 – 0.76	**0.014**	0.41	0.20	0.15 – 1.07	0.073
Age (per 1 year increase)					1.00	0.01	0.97 – 1.02	0.787	1.00	0.01	0.97 – 1.02	0.850	0.99	0.01	0.97 – 1.02	0.677	1.00	0.01	0.97 – 1.02	0.766	1.00	0.02	0.97 – 1.03	0.769
Female sex									0.70	0.24	0.36 – 1.36	0.302	0.71	0.25	0.36 – 1.40	0.330	0.80	0.28	0.40 – 1.58	0.521	0.74	0.29	0.34 – 1.57	0.442
Baseline eGFR (per 1ml/min increase)													0.99	0.01	0.97 – 1.00	0.096	0.99	0.01	0.97 – 1.00	0.111	1.00	0.01	0.98 – 1.02	0.848
Baseline systolic BP (per 1mmHg increase)																	1.00	0.01	0.98 – 1.02	0.750	1.00	0.01	0.98 – 1.02	0.811
Baseline urinary PCR (per 1mg/mmol increase)																					1.01	0.00	1.00 – 1.01	**<0.001**
AIC	214.534				216.461				217.381				216.514				211.575				188.458			
log-Likelihood	-105.267				-105.230				-104.691				-103.257				-99.787				-87.229			
