# Supplementary material for: Choroidal and retinal thinning in chronic kidney disease independently associate with eGFR decline and are modifiable with treatment
Source: Nat Commun. 2023 Dec 5;14:7720. doi: 10.1038/s41467-023-43125-1 (PMC10697963; doi:10.1038/s41467-023-43125-1)
Supplement: Supplementary file 3 — Reporting Summary [file 41467_2023_43125_MOESM3_ESM.pdf]

nature portfolio

Corresponding author(s): Neeraj Dhaun

Last updated by author(s): Oct 22, 2023

Reporting Summary

Nature Portfolio wishes to improve the reproducibility of the work that we publish. This form provides structure for consistency and transparency in reporting. For further information on Nature Portfolio policies, see our Editorial Policies and the Editorial Policy Checklist.

Statistics

For all statistical analyses, confirm that the following items are present in the figure legend, table legend, main text, or Methods section.

n/a	Confirmed	
☐	☒	The exact sample size (*n*) for each experimental group/condition, given as a discrete number and unit of measurement
☒	☐	A statement on whether measurements were taken from distinct samples or whether the same sample was measured repeatedly
☐	☒	The statistical test(s) used AND whether they are one- or two-sided<br>*Only common tests should be described solely by name; describe more complex techniques in the Methods section.*
☐	☒	A description of all covariates tested
☐	☒	A description of any assumptions or corrections, such as tests of normality and adjustment for multiple comparisons
☐	☒	A full description of the statistical parameters including central tendency (e.g. means) or other basic estimates (e.g. regression coefficient) AND variation (e.g. standard deviation) or associated estimates of uncertainty (e.g. confidence intervals)
☐	☒	For null hypothesis testing, the test statistic (e.g. *F*, *t*, *r*) with confidence intervals, effect sizes, degrees of freedom and *P* value noted<br>*Give P values as exact values whenever suitable.*
☒	☐	For Bayesian analysis, information on the choice of priors and Markov chain Monte Carlo settings
☒	☐	For hierarchical and complex designs, identification of the appropriate level for tests and full reporting of outcomes
☐	☒	Estimates of effect sizes (e.g. Cohen's *d*, Pearson's *r*), indicating how they were calculated

Our web collection on statistics for biologists contains articles on many of the points above.

Software and code

Policy information about availability of computer code

Data collection	No software was used
Data analysis	Statistical analysis was performed using Prism version 8.2.1 (GraphPad Software Inc) and R version 3.6.1 (R Foundation).

For manuscripts utilizing custom algorithms or software that are central to the research but not yet described in published literature, software must be made available to editors and reviewers. We strongly encourage code deposition in a community repository (e.g. GitHub). See the Nature Portfolio guidelines for submitting code & software for further information.

Data

Policy information about availability of data

All manuscripts must include a data availability statement. This statement should provide the following information, where applicable:

- Accession codes, unique identifiers, or web links for publicly available datasets
- A description of any restrictions on data availability
- For clinical datasets or third party data, please ensure that the statement adheres to our policy

Source data have been supplied with the manuscript

Research involving human participants, their data, or biological material

Policy information about studies with human participants or human data. See also policy information about sex, gender (identity/presentation), and sexual orientation and race, ethnicity and racism.

Reporting on sex and gender	Sex was determined by self reporting. Results apply to both sexes. Consent was not obtained for disaggregated analysis.
Reporting on race, ethnicity, or other socially relevant groupings	Ethnicity and race data were not collected.
Population characteristics	Human adult (>18 years of age) participants who were either healthy or had a chronic kidney disease including a kidney transplant.
Recruitment	Patients were recruited by established research team integrated within department of nephrology. Verbal and written information about the study was provided and with consent obtained at a subsequent visit. This may have selected patients who were engaged with nephrology services and healthcare in general. Observations may not therefore be fully representative of all typical patients with chronic kidney disease. Healthy volunteers recruited through advertisement and locally approved registers. Verbal and written information about the study was provided and with consent obtained at a subsequent visitThis may have selected a particularly healthy control population given their engagement with health research.
Ethics oversight	All studies were carried out at the University of Edinburgh according to the principles of the Declaration of Helsinki. They were approved by the South East Scotland Research Ethics committee and ACCORD and were performed with written informed consent from each subject.

Note that full information on the approval of the study protocol must also be provided in the manuscript.

Field-specific reporting

Please select the one below that is the best fit for your research. If you are not sure, read the appropriate sections before making your selection.

- [x] Life sciences
- [ ] Behavioural & social sciences
- [ ] Ecological, evolutionary & environmental sciences

For a reference copy of the document with all sections, see nature.com/documents/nr-reporting-summary-flat.pdf

Life sciences study design

All studies must disclose on these points even when the disclosure is negative.

Sample size

Study 1
The primary endpoint of this study was difference in choroidal thickness between CKD patients and healthy controls. Secondary endpoints included difference in retinal and RNFL thickness. This study was powered on the basis of a healthy subfoveal choroidal thickness of 289±52 μm and a healthy temporal RNFL thickness of 79±16 μm.27,28 To detect a difference of 10% in choroidal thickness between healthy controls and patients with CKD, or in those with a functional kidney transplant, with 80% power and a two-sided significance of 5%, we calculated that we would need to recruit 82 participants in each group. To detect a similar magnitude of change in RNFL thickness, we would require 63 subjects in each group.

Study 2: OCT metrics and histological kidney injury
The primary endpoint of this study was association between choroidal thickness and kidney scarring reflected by extent of glomerulosclerosis and interstitial fibrosis and tubular atrophy. This study was powered based on our previous work, with 40 patients providing 80 percent probability to detect a relationship between kidney scarring and choroidal thickness at two-sided significance of 5%, assuming a standard deviation of choroidal thickness of ±50um.

Study 3: OCT metrics and change in acute GFR change
The primary endpoint was a difference in OCT metrics 12 months after surgery compared to pre-surgery. The studies were designed to detect differences in OCT metrics with 80% power and with a two-sided significance of 5%. Kidney transplant recipients: our previous work in patients with kidney failure showed a choroidal thickness of 241±50 μm and an RNFL thickness of 48±11 μm.21 To detect a 15% change in choroidal thickness in patients with kidney failure undergoing kidney transplantation, we would need 24 subjects. To detect the same change in RNFL thickness, 19 subjects would be required. Kidney donors: to detect a 15% change in choroidal thickness in a healthy subject pre- and post-kidney donation, we needed to recruit 21 subjects. To detect a similar magnitude of change in RNFL thickness, 20 subjects were needed.

Study 4: Association of OCT metrics with eGFR decline in patients with pre-dialysis CKD
For this study, our primary outcomes were a decline in eGFR of ≥10% at one year and ≥20% at two years. Linked, routinely collected, individual-level biochemistry data were accessed from our regional registry of renal patients, VitalData. The 'index' or baseline eGFR was defined as the first test result available within six months of the OCT scan. One-year outcomes were calculated based on the first available measure of eGFR during a six-month window one year following the index eGFR. Two-year outcomes were calculated based on the first available measure of eGFR during a six-month window one year following the result used to define the one-year outcome. The study endpoints were selected based on the results of a meta-analysis of >1.7 million patients, which demonstrated that reductions in eGFR such as these were frequently and robustly associated with subsequent risk of kidney failure in patients with and without renal impairment at baseline.29 From our previous work,21,30 we estimated that the standard deviation of baseline eGFR in our population would be ~25 mL/min/1.73m2 and that the difference in mean eGFR at one year would be ~5 mL/min/1.73m2. Therefore, with a sample size of ~220 and an

	alpha of 0.05, our study would have a power of at least 80% to detect our pre-specified endpoints at one and two years.
Data exclusions	None
Replication	Not relevant in clincial study where individuals were studied in single point in time.
Randomization	Not relevant, observation studies
Blinding	Investigators performing and analysing OCT scans and assessing kidney biopsy specimens were blinded to clinical data.

Reporting for specific materials, systems and methods

We require information from authors about some types of materials, experimental systems and methods used in many studies. Here, indicate whether each material, system or method listed is relevant to your study. If you are not sure if a list item applies to your research, read the appropriate section before selecting a response.

Materials & experimental systems

n/a	Involved in the study	
☒	☐	Antibodies
☒	☐	Eukaryotic cell lines
☒	☐	Palaeontology and archaeology
☒	☐	Animals and other organisms
☐	☒	Clinical data
☒	☐	Dual use research of concern
☒	☐	Plants

Methods

n/a	Involved in the study	
☒	☐	ChIP-seq
☒	☐	Flow cytometry
☒	☐	MRI-based neuroimaging

Clinical data

Policy information about clinical studies

All manuscripts should comply with the ICMJEguidelines for publication of clinical research and a completedCONSORT checklist must be included with all submissions.

Clinical trial registration	The OCT And NEphropathy (OCTANE) study is registered at ClinicalTrials.gov: NCT02132741.
Study protocol	Available at ClinicalTrials.gov: NCT02132741 and from request from corresponding author
Data collection	Collected at University of Edinburgh.Subjects were recruited during August 2016 and September 2020.
Outcomes	Study 1: OCT metrics in health, CKD and kidney transplantation<br>The primary endpoint of this study was difference in choroidal thickness between CKD patients and healthy controls. Secondary endpoints included difference in retinal and RNFL thickness. This study was powered on the basis of a healthy subfoveal choroidal thickness of 289±52 µm and a healthy temporal RNFL thickness of 79±16 µm.27,28 To detect a difference of 10% in choroidal thickness between healthy controls and patients with CKD, or in those with a functional kidney transplant, with 80% power and a two-sided significance of 5%, we calculated that we would need to recruit 82 participants in each group. To detect a similar magnitude of change in RNFL thickness, we would require 63 subjects in each group. Primary and secondary outcomes were assessed by comparing means of choroidal and retinal thicknesss between three groups.<br><br>Study 2: OCT metrics and histological kidney injEndpoints were assessed examining associations between choroidal thickness or macular volume and the subsequent decline in kidney function using multiple logistic regression models adjusted for other factors that could contribute to kidney function decline.ury<br>The primary endpoint of this study was association between choroidal thickness and kidney scarring reflected by extent of glomerulosclerosis and interstital fibrosis and tubular atrophy. This study was powered based on our previous work, with 40 patients providing 80 percent probability to detect a relationship between kidney scarring and choroidal thickness at two-sided significance of 5%, assuming a standard deviation of choroidal thickness of ±50um. There were no additional secondary outcomes. End points were assessed by correlation coefficients between OCT and kidney histology metrics.<br><br>Study 3: OCT metrics and change in acute GFR change<br>The primary endpoint was a difference in OCT metrics 12 months after surgery compared to pre-surgery. The studies were designed to detect differences in OCT metrics with 80% power and with a two-sided significance of 5%. Kidney transplant recipients: our previous work in patients with kidney failure showed a choroidal thickness of 241±50 µm and an RNFL thickness of 48±11 µm.21 To detect a 15% change in choroidal thickness in patients with kidney failure undergoing kidney transplantation, we would need 24 subjects. To detect the same change in RNFL thickness, 19 subjects would be required. Kidney donors: to detect a 15% change in choroidal thickness in a healthy subject pre- and post-kidney donation, we needed to recruit 21 subjects. To detect a similar magnitude of change in RNFL thickness, 20 subjects were needed. Primary and secondary endpoints were assessed by examining OCT metrics at routine clinical time points following kidney transplantation and donation compared to values prior to surgery.<br><br>Study 4: Association of OCT metrics with eGFR decline in patients with pre-dialysis CKD

For this study, our primary outcomes were a decline in eGFR of ≥10% at one year and ≥20% at two years. Linked, routinely collected, individual-level biochemistry data were accessed from our regional registry of renal patients, VitalData. The 'index' or baseline eGFR was defined as the first test result available within six months of the OCT scan. One-year outcomes were calculated based on the first available measure of eGFR during a six-month window one year following the index eGFR. Two-year outcomes were calculated based on the first available measure of eGFR during a six-month window one year following the result used to define the one-year outcome. The study endpoints were selected based on the results of a meta-analysis of >1.7 million patients, which demonstrated that reductions in eGFR such as these were frequently and robustly associated with subsequent risk of kidney failure in patients with and without renal impairment at baseline.29 From our previous work,21,30 we estimated that the standard deviation of baseline eGFR in our population would be ~25 mL/min/1.73m2 and that the difference in mean eGFR at one year would be ~5 mL/min/1.73m2. Therefore, with a sample size of ~220 and an alpha of 0.05, our study would have a power of at least 80% to detect our pre-specified endpoints at one and two years. Endpoints were assessed examining associations between choroidal thickness or macular volume and the subsequent decline in kidney function using multiple logistic regression models adjusted for other factors that could contribute to kidney function decline.
